# Burden of chronic skin disease from an Asian perspective: Assessment of health state utilities and quality of life in a Singapore cohort

**DOI:** 10.1016/j.jdin.2024.07.020

**Published:** 2024-08-23

**Authors:** Yik Weng Yew, John Barbieri, Suephy C. Chen

**Affiliations:** a Department of Dermatology, National Skin Centre, Singapore, Singapore; b Population & Global Health Division, Lee Kong Chian School of Medicine, Nanyang Technological University, Clinical Sciences Building, Singapore, Singapore; c Department of Dermatology, Brigham and Women’s Hospital, Boston, Massachusetts; d Department of Dermatology, Duke University School of Medicine and Durham VA Medical Center, Durham, North Carolina

**Keywords:** disease burden, health state utilities, quality of life

## Abstract

**Background:**

Understanding health state utilities of skin diseases is essential for health economic evaluations in an era of rising health care costs.

**Objective:**

To create a catalog of utility values of chronic skin diseases among Asians.

**Methods:**

This is a cross-sectional study of adults attending a dermatology outpatient clinic from February 2019 to March 2023 with one of the following skin diseases: (1) eczema, (2) psoriasis, (3) acne vulgaris, (4) chronic urticaria, (5) pigmentary disorders, (6) hair loss, (7) viral warts, (8) fungal infections, and (9) keloids. Demographic and socioeconomic information was collected. Health status and utilities (Dermatology Life Quality Index, Skindex-16, EuroQol-5 Dimension, visual analog scale, time-trade-off, and willingness-to-pay) were measured.

**Results:**

A total of 183 patients with a median age of 35.5 (21-77 years) years were included. Majority (76.9%) were Chinese, followed by Malays (11.5%). The time-trade-off utilities were lower than EuroQol-5 Dimension utilities across all disease severity and most skin diseases. Patients were willing to pay $740 USD or more for a hypothetical drug to cure conditions such as psoriasis, acne, hair loss, and keloids.

**Limitations:**

Limited sample size and ethnic representation.

**Conclusions:**

This study provides a catalog of utilities in skin diseases and highlights the strengths and challenges of different measures.

**Capsule Summary**

• Our understanding of health state utilities of skin diseases is incomplete; however, this is important in an era of rising health care costs.
• Use of EuroQol5D (EQ-5D) Dimension derived utility measures may underestimate the burden of skin diseases when compared with skin-specific measures. A better, simple yet accurate measure is needed.

## Introduction

With rising health care costs, health economic evaluations are important to understand which treatments provide the highest value, particularly with development of expensive therapies such as biologics.^1^ These evaluations are an important aspect of health care policy and coverage decisions in a variety of countries, such as Canada, the United Kingdom, and Singapore.^2^^,^^3^ Health utility assessment is a key input to health economic evaluations. Utilities reflect an individual’s preferences for a particular health outcome and range from 0 to 1 (death to perfect health).^4^^,^^5^

However, little is known about utilities for common skin diseases and how these can be measured most effectively.^4^ Utilities may be sensitive to one’s age, gender, and socioeconomic status.^6^ For example, for a younger patient, the health state of a prominent shoulder keloid may be closer to a preference for death than that for the older patient, who does not care as much for appearances.

Data on quality of life (QOL) and health state utilities of skin diseases are mainly from the Western world, and an Asian perspective is often lacking. Singapore, with her diverse Asian populations, provides the unique opportunity to assess the utilities of skin diseases in Asia. We therefore aimed to create a catalog of utilities values to measure the burden of chronic skin diseases among an Asian clinical cohort. In addition, we seek to compare several assessment strategies for health economic evaluations, including time trade-off, willingness-to-pay (WTP), and the EuroQol-5 Dimension (EQ-5D).

## Methods

This is a prospective study to evaluate the QOL and health utilities of 9 skin conditions based on the 2010 WHO Global Burden of Skin Disease (GBS) study among patients attending the National Skin Centre (NSC).

### Skin diseases represented

We focus on the top skin conditions identified at the 2010 WHO Burden of Skin Disease study.^1^ The following 9 conditions included are: (1) eczema, (2) psoriasis, (3) acne vulgaris, (4) chronic urticaria, (5) pigmentary disorders, (6) hair loss, (7) viral warts, (8) fungal infections, and (9) keloids. Although keloids were not among those identified by the 2010 study, we felt that it is a common skin condition whose disfiguring nature, with its itch and pain, makes it an impactful skin condition. To ensure that each skin disease condition was adequately represented in the analysis, a target sample size of about 30 participants per disease was decided a priori. A target of 15 patients for each of the viral warts and fungal infections were made to represent nonbacterial cutaneous infections.

### Study population

To measure health state utilities among patients with preidentified skin conditions, the study population consisted of patients attending the outpatient dermatology clinic, NSC for skin conditions from February 2019 to March 2023. The NSC sees >80% of all outpatient dermatology attendances in Singapore, and therefore the patient population represented a widely distributed population in Singapore. We performed convenience sampling by enrolling patients attending the clinic consecutively. Study recruitment was interrupted during the COVID-19 pandemic, but otherwise the participation rate was >70%. The most common reason for declining participation was a lack of time. All patients recruited in the study were adults and could understand simple instructions in English to answer several questionnaire tools and instruments that have been validated in English.

Participants’ demographic information on age, gender, race, highest education level, and monthly income was collected. A 5-point Likert scale was used to rate global disease severity from both the patient’s and investigator’s perspective. Health status and utilities were measured using the Dermatology Life Quality Index (DLQI), Skindex-16, EQ-5D, visual analog scale (VAS), time-trade-off (TTO), and WTP.^5^^,^^7^, ^8^, ^9^, ^10^, ^11^

### QOL and health utilities instruments

#### DLQI

The DLQI is a widely used tool to assess the impact of skin conditions on an individual's QOL. It is a simple questionnaire that covers various aspects of daily life, including symptoms, emotions, and activities, and personal relationships affected by skin disorders. Patients were asked to fill the tool that consists of 10 questions with an overall total score range from 0 to 30, with higher scores indicating a greater impairment in QOL due to dermatologic issues.

#### Skindex-16

The Skindex-16 is a dermatology-specific QOL instrument designed to assess the impact of skin diseases on patients' lives. There are 16 items that cover 3 domains: symptoms, emotions, and functioning. Respondents rate the extent to which they have experienced specific issues related to their skin condition, with response options ranging from Never to All the time. The total score range for Skindex-16 is 0 to 100, where higher scores indicate a greater negative impact on QOL.

#### EQ-5D

The EQ-5D, is an instrument used to measure health-related QOL. Patients were asked to complete questions that encompassed 5 dimensions: mobility, self-care, usual activities, pain or discomfort, and anxiety or depression. Patients rated their health state within each dimension by choosing one of 5 levels: no problems, slight problems, moderate problems, severe problems, or extreme problems. The resulting health states were then converted into a single summary index using country-specific value sets, reflecting societal preferences for different health states. The specific value set from Malaysia was specifically used in this analysis as the societal and cultural characteristics of Singapore are closer to those of Malaysia than published indices from other countries.

#### VAS

Participants were asked to rate the severity of the health states investigated on a VAS, with 100 reflecting perfect health and 0 the worst imaginable condition.

#### TTO

Participants were asked to choose between a fixed guaranteed life duration in their current health or a shorter duration in the best possible health. The duration spent in the best possible health is varied to find the trade-off at which the individuals are indifferent between the 2 choices. The ratio of time remaining after the trade to the guaranteed life duration in current health yields the TTO utility. To separate the utility ratings for participants’ skin conditions from other comorbidities, they were asked to provide ratings of their current skin conditions independent from their comorbid conditions.

The questions or choices were asked in an interactive manner with the aid of a computer software to prevent anchoring effects. The allocated shorter duration in the best possible health was varied in a stepwise fashion until the participant was indifferent between the 2 choices of a fixed guaranteed life duration in their current health or a shorter duration in the best possible health.

#### WTP

Participants were asked in an open-ended question format about the amount of money that they would be willing to pay out-of-pocket for 2 hypothetical drugs, one for control of their skin condition and the other one for cure of their skin disease. They were asked to indicate their response in dollars and proportion of their monthly or annual household income.

For the drug for control of their skin conditions, they were asked how much they would pay out-of-pocket monthly to control their skin condition. If they stop taking the drug for control, their skin condition would recur immediately. For the drug for cure of their skin conditions, they were asked how much they would pay out-of-pocket as a one-time cost for an imaginary cure for their skin conditions. All these imaginary drugs are assumed not to have any side effects and will not be covered by their insurance. A higher WTP value would indicate a higher disease burden.

### Data analysis

All statistical analysis was performed using IBM SPSS Statistics 25. Categorical variables were summarized using counts and percentages. Continuous variables were summarized using mean with standard deviation (SD) or median with range. Correlation analysis was performed to assess the relationship between the different measures, and Spearman correlation coefficients were reported, with *r* ≤ 0.3 as poor (+), 0.3<*r*<0.7 as moderate (++), and *r* ≥ 0.7 as strong (+++). Instruments measuring similar construct would be expected to be strongly correlated. Regression analyses were also performed to assess the relationship between the health status measures (eg, DLQI, Skindex-16, and EQ-5D) and the preference-based measures (eg, TTO and WTP). Significance was assessed at a significant level of 0.05.

All participants provided informed consent to participate in the study. It was reviewed and approved by the institution ethics review board (National Healthcare Group Domain Specific Review Board (DSRB)-2016/00923).

## Results

### Patient population

A total of 241 patients participated in the study. Fifty-eight participants gave a TTO score of 0, implying that their skin condition would be equivalent to death. As there was a concern if they understood the concept of the TTO exercise properly, they were excluded from further analysis. Responses from the remaining 183 patients were analyzed. The median age was 35.5 years old (range, 21-77 years) and most (60%) participants were men. The majority (76.9%) of patients were Chinese, followed by Malays (11.5%) and Indians (8.2%). More than two-thirds rated their skin disease as moderate to severe (Table I).

**Table I:** Characteristics of patients with different skin conditions

Characteristics	Total	Acne	Eczema	Hair loss	Keloids	Pigmentation	Psoriasis	Tinea	Urticaria	Warts
	(*n* = 183)	(*n* = 24)	(*n* = 23)	(*n* = 20)	(*n* = 22)	(*n* = 23)	(*n* = 23)	(*n* = 13)	(*n* = 23)	(*n* = 12)
Age (y), mean	38.1	26.1	35.3	37.8	35.2	46.5	43.2	40.8	43.7	35.2
Gender (%)										
Female	39.3	25.0	21.7	30.0	40.9	78.3	34.9	15.4	56.5	41.7
Ethnic groups (%)										
Chinese	76.9	79.2	73.9	80.0	63.6	82.6	73.9	69.2	95.5	66.7
Malay	11.5	12.5	17.4	10.0	18.2	0.0	17.4	7.7	4.5	16.7
Indian	8.2	8.3	8.7	10.0	9.1	13.0	4.3	15.4	0.0	8.3
Others	3.3	0.0	0.0	0.0	9.1	4.3	4.3	7.7	0.0	8.3
Educational level (%)										
Primary school and below	1.6	0.0	0.0	0.0	0.0	0.0	13.0	0.0	0.0	0.0
O-level/ITE	22.4	12.5	26.1	15.0	18.2	21.7	43.5	15.4	26.1	16.7
A-level/diploma	32.8	37.5	39.1	35.0	27.3	39.1	13.0	53.8	26.1	33.3
Undergrad/Postgrad	43.2	50.0	34.8	50.0	54.5	39.1	30.4	30.8	47.8	50.0
Household income level (%)										
<$1000	7.7	16.7	8.7	0.0	0.0	4.23	8.7	7.7	13.0	8.3
$1001-$2000	9.4	4.2	13.0	10.5	14.3	8.7	17.4	0	0.0	16.7
$2001-$3500	14.9	16.7	13.0	21.1	14.3	13.0	26.1	7.7	8.7	8.3
$3501-$5000	19.9	4.2	13.0	21.1	23.8	13.0	30.4	23.1	30.4	25.0
>$5000	33.7	33.3	30.4	31.6	38.1	43.5	13.0	46.2	43.5	25.0
Unwilling to disclose	14.4	25.0	21.7	15.8	9.5	17.4	4.3	15.4	4.3	16.7
Patient global assessment (%)										
No problem	4.7	8.7	0.0	0.0	0.0	4.3	0.0	15.4	5.0	16.7
Mild	25.7	0.0	17.6	42.1	33.3	26.1	26.1	38.5	25.0	33.3
Moderate	43.9	47.8	52.9	42.1	38.1	52.2	43.5	23.1	50.0	33.3
Severe	22.8	39.1	17.6	10.5	28.6	17.4	30.4	15.4	20.0	16.7
Extremely severe	2.9	4.3	11.9	5.3	0.0	0.0	0.0	7.7	0.0	0.0
Physician global assessment (%)										
No problem	1.7	0.0	0.0	0.0	0.0	0.0	0.0	15.4	0.0	8.3
Mild	39.1	34.8	34.8	52.6	30.0	52.2	26.1	46.2	30.4	58.3
Moderate	43.0	43.5	47.8	31.6	60.0	43.5	39.1	23.1	52.2	33.3
Severe	16.2	21.7	17.4	15.8	10.0	4.3	34.8	15.4	17.4	0.0
Extremely severe	0.0	0.0	0.0	0.0	0.0	0.0	0.0	0.0	0.0	0.0

*ITE*, Institute of Technical Education.

### Health status QOL measures

The DLQI and Skindex-16 total scores in this clinical cohort ranged from 0 to 29 and 0 to 96, respectively. The Skindex-16 scores followed a normal distribution closely. There were no floor and ceiling effects for both DLQI (6.6% and 0%, respectively) and Skindex-16 (1.7% and 0%, respectively) total score. There were higher mean DLQI and Skindex-16 scores among patients with more severe skin conditions, across increasing severity banding of both patient and physician rated severity (Table II). Eczema and psoriasis have the highest DLQI and Skindex-16 scores, suggesting a worse QOL (Table III). The VAS scores ranged from 25 to 100. The data demonstrated a significant linear trend of VAS scores with increasing severity of skin diseases by both patient and physician rated global scale of severity (*P* = .003 and *P* < .001). There were lower mean VAS scores among patients with more severe skin conditions, across increasing severity banding of both patient and physician rated severity. Correlations of QOL measures with VAS and utilities were moderate to strong.

**Table II:** Health state measures across skin diseases severity

Measures (mean values)	DLQI	Skindex-16	VAS	EQ-5D	TTO	WTP-one off ($)	WTP/mo ($)
Patient global assessment (%)							
No problem	1.00	7.86	87.25	0.97	0.99	263.33	121.67
Mild	4.64	28.71	79.61	0.91	0.87	2605.56	281.22
Moderate	6.93	39.27	78.46	0.91	0.81	2667.93	242.42
Severe	12.18	61.17	71.67	0.78	0.74	2764.29	182.36
Extremely severe	16.40	71.80	63.00	0.75	0.74	14,275.00	1780.00
Physician global assessment (%)							
No problem	1.00	1.33	78.33	0.91	1.00	336.67	158.33
Mild	6.11	36.58	80.30	0.91	0.87	1641.67	245.32
Moderate	6.88	40.19	78.11	0.89	0.80	2739.86	211.23
Severe	14.66	65.29	65.59	0.75	0.71	4547.83	511.05
Extremely severe	-	-	-	-	-	-	-
Total	7.54	41.02	77.17	0.88	0.82	2887.32	272.94

*DLQI*, Dermatology Life Quality Index; *EQ-5D*, EuroQol-5 Dimensionl; *TTO*, time-trade-off; *VAS*, visual analog scale; *WTP*, willingness-to-pay.

**Table III:** Comparison of health state measures across various skin diseases

Measures (mean values)	DLQI (*n* = 183)	Skindex-16 (*n* = 176)	VAS (*n* = 183)	EQ-5D (*n* = 183)	TTO (*n* = 183)	WTP-one off ($) (*n* = 164)	WTP/mo ($) (*n* = 164)	Physical global assessment (severity) (*n* = 179)
Acne (*n* = 24)	7.63	47.26	80.38	0.89	0.77	832.50	96.20	2.87
Eczema (*n* = 23)	10.91	52.65	72.20	0.86	0.76	444.00	68.08	2.83
Hair loss (*n* = 20)	7.00	37.05	78.50	0.92	0.72	801.66	64.75	2.63
Keloids (*n* = 22)	5.41	38.86	81.36	0.90	0.76	740.00	65.12	2.80
Pigmentation (*n* = 23)	4.78	32.23	80.22	0.92	0.88	348.86	55.50	2.52
Psoriasis (*n* = 23)	10.17	45.13	69.39	0.83	0.77	1036.00	60.13	3.09
Tinea (*n* = 13)	8.15	35.50	73.23	0.80	0.92	370.00	37.00	2.38
Urticaria (*n* = 23)	8.04	45.14	80.87	0.89	0.97	416.25	59.20	2.87
Viral warts (*n* = 12)	6.58	37.45	77.50	0.86	0.83	236.80	55.50	2.25
Total	7.69	42.02	77.23	0.88	0.82	573.50	62.71	2.74

*DLQI*, Dermatology Life Quality Index; *EQ-5D*, EuroQol-5 Dimensionl; *TTO*, time-trade-off; *VAS*, visual analog scale; *WTP*, willingness-to-pay.

### Utilities measures

The EQ-5D utility summary index ranged from 0.19 to 1.00, whereas TTO utilities ranged from 0.05 to 1.00. The proportions of patients having a maximum utility score of 1.00 by EQ-5D and TTO methods were 34.4% and 54.6%, respectively. The TTO utilities were generally higher than EQ-5D utilities.

Mean EQ-5D and TTO utilities were lower across worsening patient and physician rated severity, suggesting discriminative validity (Tables II, IV, and V). However, TTO utilities were lower than EQ-5D utilities across all disease severity. Most skin conditions except tinea and urticaria also had a lower TTO than EQ-5D utilities (Table II). The correlations of EQ-5D utilities with QOL measures were moderate, whereas those of TTO utilities with QOL measures were weak (Table VI).

**Table IV:** Catalog of skin diseases utilities by TTO method by physician global assessment of severity

Skin conditions	No problem	Mild	Moderate	Severe	Very severe
Acne (*n* = 24)	-	0.68	0.81	0.8	-
Eczema (*n* = 23)	-	0.8	0.8	0.55	-
Hair loss (*n* = 20)	-	0.88	0.68	0.37	-
Keloids (*n* = 22)	-	1.00	0.64	0.5	-
Pigmentation (*n* = 23)	-	1.00	0.75	0.8	-
Psoriasis (*n* = 23)	-	0.72	0.83	0.75	-
Tinea (*n* = 13)	1.00	0.83	1.00	0.95	-
Urticaria (*n* = 23)	-	0.97	1.00	0.88	-
Viral warts (*n* = 12)	1.00	0.86	0.75	-	-
Total	1.00	0.87	0.8	0.71	-

*TTO*, Time-trade-off.

**Table V:** Catalog of skin diseases utilities by TTO method by patient global assessment of severity

Skin conditions	No problem	Mild	Moderate	Severe	Very severe
Acne (*n* = 24)	1.00	0.97	0.74	0.74	1.00
Eczema (*n* = 23)	-	0.86	0.84	0.84	0.65
Hair loss (*n* = 20)	-	0.92	0.71	0.71	0.50
Keloids (*n* = 22)	-	0.82	0.64	0.64	-
Pigmentation (*n* = 23)	1.00	0.83	0.94	0.94	-
Psoriasis (*n* = 23)	-	0.80	0.71	0.71	-
Tinea (*n* = 13)	1.00	0.98	1.00	1.00	0.90
Urticaria (*n* = 23)	1.00	0.78	0.95	0.95	-
Viral warts (*n* = 12)	0.95	0.87	0.88	0.88	-
Total	0.99	0.97	0.81	0.81	0.74

*TTO*, Time-trade-off.

**Table VI:** Correlations of health state measures

Spearman's rho *P* value	DLQI (*n* = 183)	Skindex-16 (*n* = 176)	VAS (*n* = 183)	EQ-5D (*n* = 183)	TTO (*n* = 183)	WTP-one off ($) (*n* = 164)	WTP/mo ($) (*n* = 164)
DLQI		0.822	−0.425	−0.561	−0.207	0.134	0.113
		*P* = *2.60E-44*∗	*P* = *2.05E-9*∗	*P* = *1.43E-16*∗	*P* = .005	*P* = .091	*P* = .150
Skindex-16	0.822		−0.415	−0.561	−0.136	0.146	0.044
	*P =* 2.60E-44∗		*P =* 1.02E-8∗	*P =* 5.36E-16∗	*P* =.072	*P* =.069	*P* =.582
VAS	−0.425	−0.415		0.470	0.148	−0.247	.023
	*P* = 2.05E-9∗	*P =* 1.02E-8∗		*P =* 1.98E-11∗	*P =* .046∗	*P =* .002∗	*P* = .770
EQ5D	−0.561	−0.561	0.470		0.165	−0.194	0.014
	*P = 1.43E-16*∗	*P = 5.36E-16*∗	*P = 1.98E-11*∗		*P = .026*∗	*P = .014*∗	*P* = .860
TTO	−0.207	−0.136	0.148	0.165		−0.204	−0.160
	*P* = .005	*P* = .072	*P =* .046∗	*P =* .026∗		*P =* .009∗	*P =* .041∗
WTP-one off	0.134	0.146	−0.247	−0.194	−0.204		0.556
	*P* = .091	*P* = .069	*P =* .002∗	*P =* .014∗	*P =* .009∗		*P =* 2.20E-14∗
WTP/mo	0.113	0.044	0.023	0.014	−0.160	0.556	
	*P* = .150	*P* = .582	*P* = .770	*P* = .860	*P =* .041∗	*P =* 2.20E-14∗	

*DLQI*, Dermatology Life Quality Index; *EQ-5D*, EuroQol-5 Dimensionl; *TTO*, time-trade-off; *VAS*, visual analog scale; *WTP*, willingness-to-pay.

∗ *P* < .05.

### WTP

In terms of WTP for control of their skin condition, it ranged from 0 to 1.0 and 0 to $5000/mo in terms of proportion of monthly income and absolute amount per month, respectively. For WTP to cure their skin disease, it ranged from 0 to 1.0 and 0 to $50,000 in terms of proportion of their annual income and absolute amount, respectively. There was a significant linear trend of WTP in terms of absolute amount for a cure and control of the disease with increasing severity of skin diseases by patient rated scale. (Table II) There was, however, no significant correlation of WTP with physician rated scale.

Patients were willing to pay ≥$740 USD for a hypothetical drug to cure conditions such as psoriasis, acne, hair loss, and keloids. If it was to control the skin disease, patients were willing to pay up to $96 USD per month to control acne, eczema, and keloids. The WTP measures correlated stronger and more significantly with TTO utilities when compared with EQ-5D utilities (Table III).

## Discussion

### QOL

Across all health state measures, psoriasis and eczema had the greatest impairment of health status QOL. These findings are consistent with findings from the global burden of disease study 2019 that psoriasis and eczema accounted for a total of 11 million disability-adjusted life years. These skin diseases are prevalent with a high disease burden. They often present with common symptoms of skin inflammation that can be itchy, unpleasant, and disfiguring.

### Utilities measures

Notably, TTO utilities were generally worse than EQ-5D utilities while having similar discriminative validity. TTO utilities also correlated better with WTP, another preference-based measure, compared with EQ-5D utilities and QOL measures. This was not unexpected given that TTO and WTP are preference-based measures whereas EQ-5D and QOL are health status measures. Preference-based measures consider individuals’ subjective preferences and values, whereas health status measures focus only on the objective assessment of a person’s health condition. Both types of measures measure different constructs. These findings also suggest that cost-effective analyses currently based on EQ-5Q assessment may underestimate the actual burden of skin diseases, which could influence accuracy for decision-making in health care resource allocation. Thus, the use of the EQ-5D might unfairly penalize those with skin disease if it fails to capture the health utility effects of these skin conditions and their treatment.

However, preference-based methods such as TTO can be complicated and require the participants to imagine and assess hypothetical scenarios involving both health states and duration.^12^ It may also be influenced by how the questions are asked and can be influenced by cultural and educational attainment. Therefore, there is an unmet need for better approaches to measure health utility among those with skin disease. Although there have been attempts to map EQ-5D utilities with other dermatology-specific QOL measures, such approaches will be limited by the underlying issues with respect to the content validity of the EQ-5D.^13^ Thus, there may be a need to develop novel health utility measures specific for skin disease.

### Willingness-to-pay

The WTP provides an actual quantification of the relative importance of health improvements and reflects an individual’s actual preferences for a wide range of predefined health states. However, WTP also has high subjectivity and variability. An individual’s income, value system, and personal circumstances at that moment can influence its response.^14^ Some intangible benefits and health outcomes could be difficult to quantify in monetary terms.

### Strengths and limitations

Strengths of this study include a comprehensive analysis of different skin conditions of varying severity in an Asian clinical population. This provides a useful resource to perform cost-effectiveness analyses in other similar backgrounds and settings. Various measures assessing QOL and utilities were included for comparison. However, the study has been affected by patients’ difficulties in executing the TTO tasks. Limited sample size has also prevented further stratification of analysis into ethnic, age, or social economic sub-groups. However, regression analyses adjusting for possible socioeconomic confounding factors yielded similar results. Although the study cohort included a widely distributed Singaporean population, there could be potential selection and recruitment bias given that the study cohort was a convenience sample of patients attending the clinic. Recruitment was also disrupted during the COVID-19 pandemic and affected to a certain extent patients’ access to tertiary dermatology care. Most patients were Chinese and that affected analysis among the minor ethnic groups such as Malays and Indians. However, the proportions of Chinese, Malays and Indians in the study closely mirrored that of the national population census in Singapore.^15^

Future studies should include a larger sample size and a more diverse representation of skin diseases. Further improvements and specific strategies to address the challenges in TTO tasks could be made because the correlation and prediction model for TTO from health status measures have not been good.

In summary, this study provides a catalog of utilities in chronic skin diseases in an Asian population and highlights the challenges of using preference-based measures to assess the burden of skin diseases. There is a need for a better utilities measure that are simple yet accurate in assessing skin diseases.

## Conflict of interest

Dr Barbieri has received consulting fees from Dexcel pharma for work unrelated to the present submission. Drs Yew and Chen have no conflicts of interest.
